# Genicunolide A, B and C: three new triterpenoids from *Euphorbia geniculata*

**DOI:** 10.3762/bjoc.11.291

**Published:** 2015-12-23

**Authors:** Alia Farozi, Javid Ahmad Banday, Shakeel Ahmad Shah

**Affiliations:** 1 Department of Chemistry, National Institute of Technology, Hazratbal, Srinagar-190006, J&K, India, Tel.: +91 9906626635

**Keywords:** *Euphorbiaceae*, *Euphorbia geniculata*, friedelin, friedelinol, genicunolide A, B, C, pentacyclic triterpenoids

## Abstract

Three new triterpenoids, designated as genicunolide A (**1**), B (**2**) and C (**3**), along with friedelin (**4**) and friedelinol (**5**), were isolated from the aerial parts of *Euphorbia geniculata*. They were characterized as 1β-acetoxy-3β-hydroxy-11α,12α-oxidotaraxer-14-ene, 1β,3β-diacetoxy-21α-hydroxy-11α,12α-oxidotaraxer-14-ene and 3β,9α,20α-trihydroxy-Ψ-taraxast-5-ene, respectively, by spectral and chemical methods.

## Introduction

*Euphorbia* (Euphorbiaceae) is a very large and diverse genus of flowering plants comprising of about 2,000 members and is found all over the world, ranging from short annual plants to well developed tall trees [1].

The plants of the family Euphorbiaceae contain well-known skin irritating and tumor-promoting diterpenoids with tigliane, ingenane and daphnane skeletons [2]. Some of the species are used in folk medicine to cure skin diseases, gonorrhea, migraines, intestinal parasites, and warts [3] and as a purgative [4–6]. Several macrocyclic diterpenoids with antibacterial, anticancer, anti-multidrug-resistant, antifeedant, anti-HIV and analgesic activity have been isolated from different *Euphorbia* species. They include jatrophane, ingol and myrsinane diterpenoids [7–13].

Triterpenoids which have been reported from various species of *Euphorbia* include β-amyrin [1], β-amyrin acetate [14,18], cycloeucalenol, obtusifoliol, 24-methylenecycloartan-3-β-ol, β-sitosterol, betulin, erythrodiol, oleanolic acid, β-sitosterol glucoside[15], 29-norcycloart-5-ene-5,8-lanostadiene-3β-ol, 3β,24*S*,25-trihydroxycycloartane, 3β,24(*R*),25-trihydroxy-cycloartane, 24-methylenecycloartan-3β-ol [16], cycloart-23-ene-3,5-diol [17], lupeol, lupeol acetate, ginnone, ambrein, lupeone [18], 24-methylenecycloartanol [19], cycloart-25-en-3β,24-diol [20] and cycloart-22-ene-3β,25-diol [21]. In addition, nor-isoprenoids and coumarins have also been reported from few species of *Euphorbia* [22–24].

*Euphorbia geniculata* Orteg. [25–26], is a wild weed found in the Jammu region of India [27]. The plant is locally used for the treatment of bacterial infections and inflammations. Previous phytochemical investigations have demonstrated that this plant contains flavonoids: kaempferol, quercetin and 3-rhamnosyl quercetin [28] and triterpenes β-amyrin acetate [29] and geniculatin [30].

Reinvestigation of chemistry of the plant led to isolation of three new triterpenoids, designated as genicunolide A (**1**), B (**2**) and C (**3**), together with friedelin (**4**) [31] and friedelinol (**5**) [32], from the ethyl acetate extract of the aerial parts of the plant. Herein, we report the characterization of the three compounds by spectral and chemical methods.

## Results and Discussion

**Figure 1:**
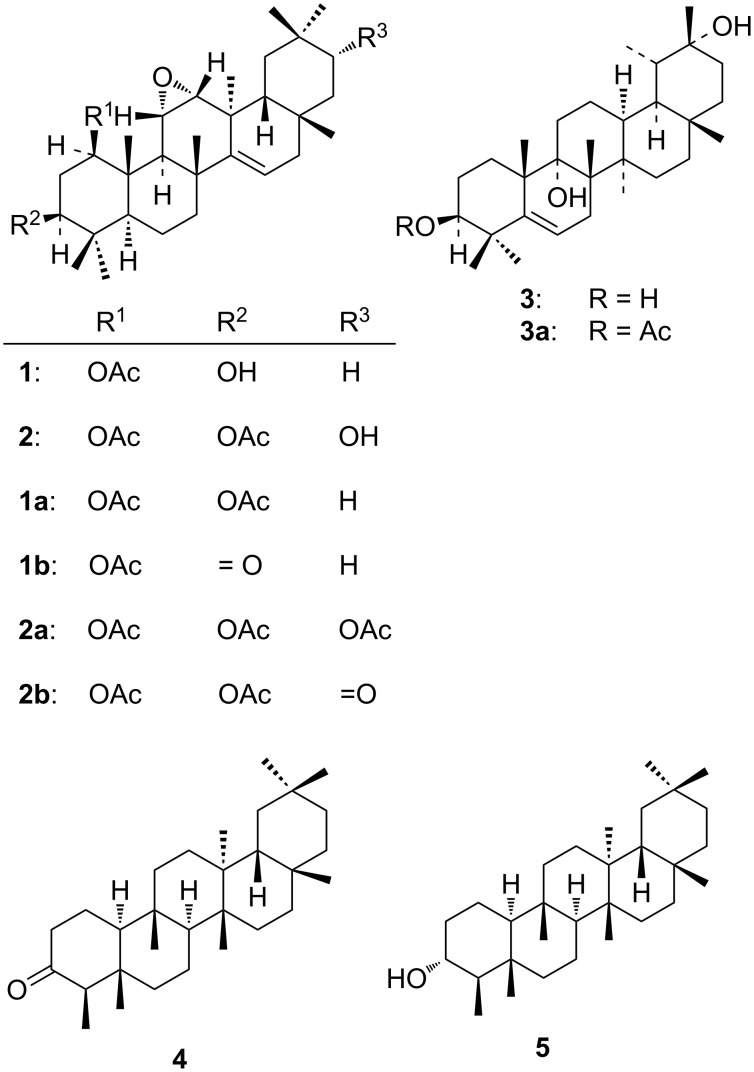
Structures of compounds **1**, **2**, **3**, **1a**, **2a**, **1b**, **2b**, **3a**, **4** and **5**.

The compounds **1**–**3** (Figure 1) responded positively to the characteristic Liebermann–Burchard [33], TCA [34–35] and TNM tests [35] for unsaturated triterpenoids.

The compound **1**, M^+^ at *m*/*z* 498.0695 (calculated for C_32_H_50_O_4_, 498.0700), possessed eight tertiary methyl groups, an acetoxy functionality [*ν_max_* 1736 cm^−1^, δ 2.03 (s, 3H), δ_C_ 170.2, 21.3], a trisubstituted double bond [*ν_max_* 1630, 1042, 880 cm^−1^, δ 5.56 (d, *J* = 5.2 Hz, 1H, H-15)] [36] and a secondary equatorial hydroxy group [ν_max_ 3500 cm^−1^] whose carbinylic proton resonated at δ 3.16 (dd, *J* = 8.1 Hz, 1H, H-3). On acetylation with Ac_2_O–C_5_H_5_N, at room temperature, compound **1** afforded the diacetate **1a**, δ 2.06 (s, 6H), and on oxidation with CrO_3_–C_5_H_5_N yielded a ketoacetate **1b**, ν_max_ 1738, 1680 cm^–1^, δ_C_ 216.7, 170.8, which responded positively to the characteristic Zimmermann test for 3-keto function [37], thereby placing the hydroxy group in compound **1** at 3β position, δ_C_ 77.2 [38].

The mass spectrum of compound **1** displayed the characteristic features of the taraxer-14-ene skeleton [39] by exhibiting RDA fragment ion peaks at *m*/*z* 374 (rings A/B/C) and 124 (ring E) and the vinylic carbon resonance signals at δ_C_ 118.8 (C-15) and 157.0 (C-14) [40].

The presence of a *cis*-oxido functionality in compound **1** was evident from a pair of AB doublets at δ 2.82 and 3.01 (*J*_ae_ = 4.7 Hz, 1H each, H_e_-12 and H_a_-11), in its ^1^H NMR spectrum, two methine carbon resonance signals at δ_C_ 53.4 (C-11) and 58.3 (C-12) [41] and loss of CO and H_2_O, via rearrangement of hydrogen [42] from the RDA fragment ion at *m*/*z* 374 to give abundant ion peaks at *m*/*z* 346 and 354, respectively.

The acetoxy functionality in compound **1** was placed at C-1 β-position on the basis of the chemical shift, multiplicity and coupling constant of the carbinylic proton [δ 4.53 (dd, *J*_ae_ = 7.4 Hz, *J*_aa_ = 8.5 Hz, 1H, H-1)] together with the identical chemical shift of H-1 and H-3, in the ^1^H NMR spectrum of **1a**, and the comparable chemical shifts of C-1 and C-3 of **1a** (δ_C_ 80.7 and 80.6, respectively) with that of 1β,3β-diacetoxylupenes [43].

The structure of compound **1** was further confirmed by ^1^H,^1^H and ^1^H,^13^C COSY, HMBC and HSQC experiments which allowed unambiguous fixation of protons to appropriate carbons and also ^13^C-chemical shifts (Table 1). The long range correlations between the protons at δ 1.96 (H_a_-2) and 1.98 (H_e_-2) and carbonyl signal at δ_C_ 170.8 confirmed the presence of acetoxy carbonyl at C-1. This was further substantiated by the long range mutual coupling of the carbinylic proton at δ 4.53 with the proton at δ 3.16 as also with the carbon at δ_C_ 41.1 ppm (C-4). The correlation cross peaks between H-23 and H-25 and H-2 in the NOESY experiment confirmed that the acetoxy function at C-1 was β-oriented. The proton at δ 2.82 was correlated to the olefinic carbon at δ_C_ 157 ppm (C-14), three bonds away and allowed joining of spin systems separated by a methyl-bearing quaternary carbon on one side. The chemical shifts, multiplicity and coupling constants of the A-ring carbinylic protons and an inspection of the molecular models suggested that 1,3-*cis*-diequatorial functions in ring A caused flattening of this ring.

**Table 1:** ^13^C NMR data of **1**, **2**, **3** and their acetates in CDCl_3_ (δ in ppm, 125 MHz).

C	**1**	**1a**	**2**	**2a**	**3**	**3a**

1	80.6	80.7	80.9	80.9	21.5	21.5
2	27.8	29.8	29.9	29.7	28.6	29.0
3	77.2	80.6	80.6	80.8	71.8	80.5
4	41.1	41.5	41.1	41.3	41.9	42.0
5	54.5	54.6	54.6	54.6	140.7	139.0
6	18.4	19.5	19.5	20.1	121.7	122.0
7	33.3	30.2	30.2	30.2	23.5	23.4
8	39.6	39.9	39.6	40.1	42.7	42.9
9	52.6	52.7	52.6	52.8	89.5	89.5
10	37.6	38.1	37.6	38.1	42.7	42.8
11	53.4	53.4	53.4	53.3	23.4	23.5
12	58.2	58.3	58.2	58.4	26.4	26.4
13	36.5	36.4	36.4	36.4	39.7	39.8
14	157.1	157.1	157.2	157.1	36.9	37.0
15	118.9	118.9	118.9	118.8	33.9	34.0
16	35.7	35.9	35.7	35.8	31.9	31.8
17	35.4	35.8	35.8	35.8	32.3	32.5
18	48.1	48.5	48.1	48.6	56.1	56.3
19	40.2	42.1	41.5	42.1	46.2	46.1
20	28.7	28.8	31.9	27.5	77.2	77.3
21	35.7	35.9	77.2	80.1	31.9	42.5
22	35.6	35.7	39.6	35.1	42.7	42.5
23	27.0	27.1	27.0	27.2	31.9	31.8
24	17.0	16.5	16.7	16.7	29.3	29.5
25	16.6	16.5	16.5	27.5	20.3	20.3
26	27.0	27.5	27.8	30.1	19.8	19.9
27	30.2	30.2	30.2	29.8	12.1	12.3
28	29.9	29.8	29.9	29.9	23.1	23.2
29	33.6	33.1	33.3	33.3	20.0	20.1
30	19.5	19.5	19.5	19.5	36.1	36.2
1-OAc	170.8, 21.3	170.7, 21.2	170.8, 21.1	170.8, 21.1	–	–
3-OAc	–	170.9, 21.4	170.9, 21.2	170.9, 21.2	–	170.5, 21.4
21-OAc	–	–	–	170.1, 21.3	–	–

The spectral patterns of compound **2**, M^+^ at *m*/*z* 556.0739, C_34_H_52_O_6_, resembled closely with those of **1a**, except that it was shown to possess an extra secondary hydroxy group [*ν_max_* 3350 cm^–1^, δ 3.17 (dd, *J* = 11.1 Hz, 1H)]. Its presence was confirmed by acetylation of **2** to **2a** [δ 2.05 (s, 9H), δ_C_ 170.1, 170.7 and 170.8] and oxidation to diacetoxy ketone **2b** [*ν_max_* 1736, 1730, 1680 cm^–1^, δ_C_ 170.5, 170.6, 215.2 (C-21)]. The mass spectrum of compound **2** exhibited RDA fragment ions at *m*/*z* 416 and 140, placing the hydroxy group in ring E. The hydroxy group was placed at C-21 α-position (δ_C_ 77.2) [38] on the basis of coupling constant of carbinylic proton in the ^1^H NMR spectrum of **2**, the downfield chemical shift of C-30 methyl protons (δ 1.09) in the spectrum of **2b**, and ^1^H,^1^H, ^1^H,^13^C COSY, HMBC and HSQC spectra of **2** which showed long range correlations of the carbinylic proton at δ 3.17 (H-21) and methyl protons at δ 0.87 (H-29) as also carbons at δ_C_ 35.7 (C-16) and 48.1 (C-18).

The ^1^H NMR, ^13^C NMR (Table 1) and DEPT (135°) spectra of compound **3**, M^+^ at *m*/*z* 458.1495, C_30_H_50_O_3_, revealed that the compound possesses seven tertiary methyls, one of which resonated downfield at δ 1.53; a secondary methyl [δ 0.85 (d, *J* = 4.2 Hz, 3H)], a trisubstituted double bond [ν_max_ 1640, 1040, 890 cm^−1^, δ 5.36 (d, *J* = 4.7 Hz, 1H)], and a secondary hydroxy group [ν_max_ 3465 cm^−1^, δ 3.52 (dd, *J*_aa_ = 7.6 Hz, H-3, 1H)]. On acetylation with Ac_2_O–C_5_H_5_N, at room temperature, it afforded monoacetate **3a** [1735, δ 2.05 (s, 3H), δ_C_ 170.5] and on oxidation with CrO_3_–C_5_H_5_N, it yielded a ketone which gave a positive Zimmermann test for 3-keto group [37] confirming the presence of the C-3 equatorial secondary hydroxy group [δ_C_ 71.8] in **3**. The mass spectrum of compound **3** revealed that the double bond triggered the typical RDA fragmentation of ring B [39] to give densely populated ion peaks at *m*/*z* 166 (ring A) and 292 (rings C/D/E) placing the double bond at C-5 [δ_C_ 122.0 (C-6), 139.9 (C-5)] [38] and two hydroxy groups in rings C/D/E. Since the monoacetate **3a** still retained a hydroxy group and its mass spectrum also showed a RDA fragment ion at *m*/*z* 292, compound **3**, therefore, carried two tertiary hydroxy groups on rings C/D/E. The presence of a secondary methyl group together with a pair of doublets at δ 1.56 and 1.85 (d br, *J* = 10.0 Hz, 1H each, H-18 and H-19) showed that the compound belonged to the Ψ-taraxastane [35] series. The downfield shift of C-30 methyl singlet (δ 1.53) suggested that one of the tertiary hydroxy groups was at C-20 (δ_C_ 77.3). Had it been on C-19, the ^13^C signal would have been observed upfield at δ_C_ 73.0–73.2 [44]. The densely populated ion peaks at *m/z* 221 (rings A/B) and 203 (221 − H_2_O**^.^**^+^), arising from the fission of 9, 11 and 8, 14 bonds in ring C, together with the downfield carbon signal at δ_C_ 89.5 placed the second tertiary hydroxy groups at C-9. The structure of compound **3** was further confirmed by ^1^H,^1^H, ^1^H,^13^C COSY and long range ^1^H,^13^C COSY experiments. The presence of the C-9 hydroxy group was proved by linking the carbon signal at δ_C_ 89.5 to proton signals at δ 1.01 (C-25) and 0.93 (C-26) in the ^1^H,^13^C long-range coupled spectrum. Other data for 1D and 2D NMR spectra of **3** were in agreement with the assigned structure.

Comparison of physical characteristics and spectral data of compounds **4** and **5**, with those reported in literature [31–32], confirmed them to be friedelin and friedelinol, respectively.

## Conclusion

The compounds **1**–**5** were, thus, characterized as 1β-acetoxy-3β*-*hydroxy-11α,12α-oxido-taraxer-14-ene (**1**); 1β,3β-diacetoxy-21α-hydroxy-11α,12α-oxido-taraxer-14-ene (**2**); 3β,9α,20α-trihydroxy-Ψ-taraxast-5-ene (**3**); friedelin (**4**) and friedelinol (**5**); respectively. Compounds **1**–**3** are new triterpenoids while **4** and **5** appear to have been isolated for the first time from the genus *Euphorbia*.

## Experimental

### General procedures

Melting points were determined in centigrade scale in one end open capillaries on a Büchi 570 melting point apparatus and are uncorrected. IR spectra were recorded on a Perkin-Elmer Paragon-1000 spectrophotometer or an Esquire 3000 spectrometer. ^1^H and ^13^C NMR spectra were recorded by a Bruker 500 and 125 MHz instrument using TMS as internal standard and CDCl_3_ as solvent. High-resolution mass spectra were recorded on a Bruker 400 mass spectrometer. Column chromatography was carried out with Merk silica gel (60–120 mesh). Optical rotation was measured on a Perkin-Elmer polarimeter.

### Plant material

The aerial parts of *Euphorbia geniculata* (Orteg) were collected from Jammu, (J&K, India) in July 2013. The specimen was identified by Akhtar H. Malik, Curator, Centre for Biodiversity & Taxanomy, University of Kashmir (Specimen deposited under accession No. 1850 – KASH Herbarium).

### Extraction and isolation

The shade dried aerial parts of *Euphorbia geniculata* (3.0 kg) were extracted sequentially with petroleum ether (60–80 °C), ethyl acetate and methanol in a soxhlet apparatus to afford respective extracts which were concentrated under reduced pressure. The ethyl acetate extract (40 g) was subjected to chromatography on silica gel (60–120 mesh, B.D.H.) column using graded solvent systems of petroleum ether–ethyl acetate. The fractions collected with petroleum ether–ethyl acetate (9:1), F-1; (8:2), F-2; (7:3), F-3 and ethyl acetate, F-4, whose components gave green, pink and violet colouration on TLC (silica gel G) plates, after development with cerric ammonium sulfate–H_2_SO_4_, were subjected to re-chromatography. The fraction F-1 on re-chromatography and elution with petroleum ether–dichloromethane (8:2) gave **4** (300 mg) and **5** (410 mg). The fraction F-2 on further chromatography and elution with petroleum ether–dichloromethane (7:3) and (8:4) gave two mixtures. The mixture obtained with petroleum ether–dichloromethane (8:4) was subjected to preparative TLC using petroleum ether–chloroform (19:3) as solvent system to get compound **1** (48 mg). The fraction F-3 on further chromatography and elution with petroleum ether–dichloromethane (8:2) gave compound **2** (45 mg). The fraction **F-4** on repeated chromatography using the same sequence of graded solvent systems, as for crude extract, gave compound **3** (38 mg) with petroleum ether–dichloromethane (3:7) and a mixture containing **3** and **2**, which was again resolved by preparative TLC using benzene–ethyl acetate (9:1) as solvent system.

**Genicunolide A (1):** Colourless crystals (CHCl_3_–Me_2_CO), mp 150 °C; [α]_D_^25^ +20.5° (*c* 0.50, CHCl_3_); HRMS: *m*/*z* (rel. int.) 498.0695 (18) (M^+^) (calcd for C_32_H_50_O_4_, 498.0700), 483 (36.2), 480 (21.4), 456 (28.6), 441 (47.1), 374 (71.3) (RDA, rings A/B/C), 346 (57.2), 314 (42.7), 124 (65.8) (RDA, ring E), 108 (100); IR: ν_max_ 3500 (OH), 3030, 2850, 1736 (OAc), 1630, 1456, 1042, 880 cm^−1^; ^1^H NMR (500 MHz, CDCl_3_) δ 0.80 (s, 3H, H-28), 0.86 (s, 6H, H-24, H-29), 0.89 (s, 3H, H-30), 0.90 (s, 3H, H-23), 1.01 (s, 3H, H-25), 1.09 (s, 3H, H-26), 1.25 (s, 3H, H-27), 2.06 (s, 3H, OCOCH_3_), 2.31 (d, *J* = 6.9 Hz, 2H, H-16), 2.82 (d, *J* = 4.7 Hz, 1H, H-12), 3.01 (d, *J* = 4.7 Hz, 1H, H-11), 3.16 (dd, *J* = 5.5, 8.1 Hz, 1H, H-3), 4.53 (dd, *J* = 7.4, 8.5 Hz, 1H, H- 1), 5.56 (d, *J* = 5.2 Hz, 1H, H-15); ^13^C NMR: Table 1.

**Genicunolide B (2):** Colourless crystals (CHCl_3_–Me_2_CO), mp 160 °C, [α]_D_^25^ + 34.2° (*c* 0.40, CHCl_3_); HRMS: *m*/*z* 556.0739 (M^+^) (calcd for C_34_H_52_O_6_, 556.0744), 541 (M^+^ − **^.^**CH_3_), 514 (M^+^ − CH_2_CO), 472 (514 − CH_2_CO), 454 (472 − H_2_O), 416 (RDA, rings A/B/C), 356 (416 − HOAc), 286 (356 − CO − CH_2_CO), 140, 124 (RDA, ring E), 108 (124 – H2O)(100); IR: ν_max_ 3550, 3025, 2863, 1736 (OAc), 1730 (OAc), 1625, 1450, 1045, 890 cm^−1^; ^1^H NMR (500 MHz, CDCl_3_) δ 0.80 (s, 3H, H-28), 0.87 (s, 6H, H-24, H-29), 0.97 (s, 3H, H-30), 0.98 (s, 3H, H-23), 1.01 (s, 3H, H-25), 1.09 (s, 3H, H-26), 1.25 (s, 3H, H-27), 2.06 (s, 6H, 2 x OAc), 2.31 (s, 2H, H-16), 2.80 (d, *J* = 4.7 Hz, 1H, H-12), 3.01 (d, *J* = 4.8 Hz, 1H, H-11), 3.17 (dd, *J* = 5.5, 11.1 Hz, 1H, H-21), 4.53 (dd, *J* = 7.6, 8.5 Hz, 2H, H_a_-1, H_a_-3), 5.56 (d, *J* = 5.2 Hz, 1H, H-15); ^13^C NMR: Table 1.

**Genicunolide C (3):** Colourless needles, mp 210–211 °C, [α]_D_^25^ +30.3° (*c* 0.3, CHCl_3_); HRMS: *m*/*z* 458.1495 (M**^.^**^+^) (calcd for C_30_H_50_O_3_, 458.1500) (M**^.^**^+^), 443 (M^+^ − CH_3_), 440 (M^+^ − H_2_O), 425 (M^+^ − CH_3_ − H_2_O), 413 (M^+^ − CH_3_CH=^+^OH), 292 (RDA, rings C/D/E), 237 (RDA, rings D/E), 221, 203, 166 (RDA, ring A), 163, 107, 83, 45 (100); IR: *ν_max_* 3580, 3465, 1640, 1445, 1040, 1025, 890 cm^−1^; ^1^H NMR (500 MHz, CDCl_3_) δ 0.68 (s, 3H, H-28), 0.79 (s, 3H, H-27), 0.81 (s, 3H, H-23), 0.85 (d, *J* = 4.2 Hz, 3H, H-29), 0.90 (s, 3H, H-24), 0.93 (s, 3H, H-26), 1.01 (s, 3H, H-25), 1.53 (s, 3H, H-30), 1.56 (d br, *J* = 10.0 Hz, 1H, H-18), 1.85 (d br, *J* = 10.0 Hz, 1H, H-19), 2.28 (s, 2H, H-7), 3.52 (dd, *J* = 4.8, 7.6 Hz, 1H, H-3), 5.36 (d, *J* = 4.7 Hz, 1H, H-6); ^13^C NMR: Table 1.

**Acetylation of 1, 2 and 3:** Compounds **1, 2** and **3** (20 mg each) were dissolved separately in C_5_H_5_N (2 mL) and Ac_2_O (2 mL) was added. The reaction mixtures were left overnight, diluted with water and extracted with chloroform. The chloroform solutions were washed with 5% HCl–H_2_O solution (10 mL each time) and dried over anhydrous K_2_CO_3_. After removal of the solvent, the crude acetates were purified by column chromatography on silica gel using petroleum ether–benzene (9:1, 7:3 and 1:1 v/v) when **1a**, **2a** and **3a** (18 mg, 17 mg and 19 mg, respectively) were recovered.

**Oxidation of 1 and 2:** Compound **1** (12 mg) and compound **2** (15 mg) were dissolved separately in C_5_H_5_N (1 mL) and treated with freshly prepared CrO_3_–C_5_H_5_N complex. The reaction mixtures were left overnight, diluted with water (10 mL) and extracted with chloroform (3 × 20 mL). The chloroform layer was washed with water, 0.1 N HCl, water and dried over anhydrous MgSO_4_. After removal of the solvent, the residues were purified by column chromatography over silica gel using a petroleum ether–benzene (1:1 v/v) solvent system, and crystallized from CHCl_3_–Me_2_CO.

## Supporting Information

File 1 Spectral data of genicunolide A acetate (**1a**), genicunolide B acetate (**2a**), genicunolide C acetate (**3a**), oxogenicunolide A (**1b**), oxogenicunolide B (**2b**), friedelin (**4**) and friedelinol (**5**).
