# Supplementary material for: Genicunolide A, B and C: three new triterpenoids from Euphorbia geniculata
Source: Beilstein J Org Chem. 2015 Dec 23;11:2707–12. doi: 10.3762/bjoc.11.291 (PMC4734298; doi:10.3762/bjoc.11.291)
Supplement: File 1 — Spectral data of genicunolide A acetate (1a), genicunolide B acetate (2a), genicunolide C acetate (3a), oxogenicunolide A (1b), oxogenicunolide B (2b), friedelin (4) and friedelinol (5). [file Beilstein_J_Org_Chem-11-2707-s001.pdf]

Supporting Information

for

Genicunolide A, B and C: three new triterpenoids from *Euphorbia geniculata*

Alia Farozi, Javid A. Banday* and Shakeel A. Shah

Address: Department of Chemistry, National Institute of Technology, Hazratbal, Srinagar-190006, J&K, India, Tel.: +91 9906626635

Email: Javid A. Banday* - javidbanday@rediffmail.com
*Corresponding author

Spectral data of genicunolide A acetate (1a), genicunolide B acetate (2a), genicunolide C acetate (3a), oxogenicunolide A (1b), oxogenicunolide B (2b), friedelin (4) and friedelinol (5)

Genicunolide A acetate (**1a**)

Colourless needles ($CHCl_3$-Me_2CO), m.p. 148°C, HRMS: *m/z* (rel. int.) 540.0051 (37) ($M^{\cdot+}$) (calc. for $C_{34}H_{52}O_5$, 540.0056), 525 (18.5), 498 (10.3), 438 (38.7), 423 (45.6), 416 (56.2),332 (60.0), 314 (41.5), 304 (41.3), 286 (25.5), 124 (41.5), 108 (100). IR: ν_{max} 3030, 2850, 1738, 1739, 1630, 1456, 1042, 870 cm^{-1}. ^{1}H-NMR (500 MHz, $CDCl_3$) : δ 0.80 (3H, s, H-28), 0.84 (3H, s, H-29), 0.87 (3H, s, H-24), 0.92 (3H, s, H-23), 0.94 (3H, s, H-30), 1.01 (3H, s, H-25), 1.09 (3H, s, H-26), 1.25 (3H, s, H-27), 2.06 (6H, s, 2 x $OCOCH_3$), 2.31 (2H, s br, H-16), 2.82 (1H, d, J = 4.7 Hz, H-12), 3.01 (1H, d, J = 4.7 Hz, H-11), 4.55 (2H, dd, J = 7.6, 8.4 Hz, H-1 and H-3), 5.55 (1H, d, J = 5.2 Hz, H-15). ^{13}C-NMR: Table 1.

Genicunolide B acetate (**2a**)

Colourless needles ($CHCl_3$-Me_2CO), m.p. 155^oC, HRMS: *m/z* (rel. int.) 598.1383 ($M^{\cdot+}$) (19.5) (calc. for $C_{36}H_{54}O_7$, 598.1388), 583 (9.5), 523 (16.7), 514 (13.3), 472 (10.5), 454 (23.9), 416 (38.9), 332 (52.5), 304 (67.3), 182 (46.5), 166 (31.4), 106 (100). ^{1}H-NMR (500 MHz, $CDCl_3$): δ 0.80 (3H, s, H-28), 0.85 (3H, s, H-29), 0.87 (3H, s, H-24), 0.95 (3H, s, H-30), 0.98 (3H, s, H-23), 1.01 (3H, s, H-25), 1.09 (3H, s, H-26), 1.25 (3H, s, H-27), 2.05 (9H, s, 3 x OAc), 2.30 (2H, s, H-16), 2.81 (1H, d, *J* = 4.7 Hz, H-12), 3.02 (1H, d, *J* = 4.7 Hz, H-11), 4.32 (1H, dd, *J* = 5.5, 11.1 Hz, H-21), 4.53 (2H, dd, *J* = 7.6, 8.5 Hz, H-1, H-3), 5.55 (1H, d, *J* = 5.5 Hz, H-15). ^{13}C-NMR: Table 1.

Genicunolide C acetate (**3a**)

Colourless crystals ($CHCl_3$-MeOH), m.p. 205-206^oC, HRMS: *m/z* (rel. int.) 500.0851 ($M^{\cdot+}$) (4.5) (calc. for $C_{32}H_{52}O_4$, 500.0856), 485 (13.6), 482 (31.7), 464 (12.1), 467 (33.5), 458 (23.4), 292 [RDA, rings C/D/E] (67.2), 263 [8, 14; 9-11 fission, ring A/B] (59.5), 237 (49.2), 208 [RDA, ring A] (74), 203 [263-HOAc] (85.0), 163 (76.4), 107 (63.5), 45(100). ^{1}H-NMR (500 MHz, $CDCl_3$): *δ* 0.68 (3H, s, H-28), 0.80 (3H, s, H-27), 0.82 (3H, s, H-23), 0.85 (3H, d, *J* = 4.3 Hz, H-29), 0.91 (3H, s, H-24), 0.94 (3H, s, H-26), 1.02 (3H, s, H-25), 1.55 (3H, s, H-30), 2.05 (3H, s, OAc), 2.30 (2H, d, *J* = 7.9 Hz, H-7), 4.25 (1H, dd, *J* = 4.8, 7.6 Hz, H-3), 5.38 (1H, d, *J* = 4.7 Hz, H-6). ^{13}C-NMR: Table 1.

Oxogenicunolide A (**1b**)

Colourless needles, m.p. 130^oC, HRMS: *m/z* (rel. int.) 496.0539 ($M^{\cdot+}$) (14.6) (calc. for $C_{32}H_{48}O_4$, 496.0544), 481 (37.5), 454 (93.3), 421 (59.2), 436 (30.8),372 (67.9), 316 (55.8), 312 (48.9), 256 (29.7), 124 (78.3), 108 (100). IR: ν_{max} 3030, 2860, 1738, 1680, 1625, 1456, 1042, 880 cm^{-1}. ^{1}H-NMR (500 MHz, $CDCl_3$) : *δ* 0.80 (3H, s, H-28), 0.86 (3H, s, H-29), 0.88 (3H, s, H-24), 0.89 (3H, s, H-30), 0.99 (3H, s, H-23), 1.01 (3H, s, H-25), 1.09 (3H, s, H-26), 1.25 (3H, s, H-27), 2.06 (3H, s, $OCOCH_3$), 2.31 (2H, d, J = 6.8 Hz, H-16), 2.35 (2H, m, H-2), 2.81 (1H, d, *J* = 4.7 Hz, H-12), 3.01 (1H, d, *J* = 4.7 Hz, H-11), 4.54 (1H, dd, *J* = 7.4, 8.4 Hz, H-1), 5.55 (1H, d, *J* = 5.5 Hz, H-15). ^{13}C-NMR: δ_C 16.5 (C-25), 18.5 (C-24), 19.1 (C-30), 19.2 (C-6), 21.4 ($OCOCH_3$), 27.0 (C-26), 28.1 (C-23), 28.8 (C-20), 30.1 (C-27), 30.9 (C-2), 33.1 (C-7), 33.6 (C-29), 35.6 (C-16, C-

17), 35.7 (C-22), 36.1 (C-21), 36.4 (C-13), 37.7 (C-10), 39.6 (C-8), 40.1 (C-19), 44.2 (C-4), 48.1 (C-18), 52.8 (C-9), 53.4 (C-11), 54.5 (C-5), 58.3 (C-12), 80.7 (C-1), 118.8 (C-15), 157.3 (C-14), 170.8 ($OCOCH_3$), 216.7 (C-3).

Oxogenicunolide B (**2b**)

Colourless crystals (10 mg), m.p. 133°C, HRMS: *m/z* (rel. int.) 554.0895 ($M^{\cdot+}$) (28.3) (calc. for $C_{34}H_{50}O_6$, 554.0900), 539 (10.3), 479 (67.5), 470 (32.1), 452 (23.6), 416 (57.2), 356 (55.1), 332 (40.8), 318 (32.7), 304 (57.9), 224 (27.7), 138 (80.2), 122 (100). IR: ν_{max} 3025, 2860, 1736, 1730, 1680, 1627, 1450, 1043, 890 cm^{-1}. ^{1}H-NMR (500 MHz, $CDCl_3$): δ 0.81 (3H, s, H-28), 0.87 (6H, s, H-24), 0.89 (3H, s, H-29), 0.98 (3H, s, H-23), 1.01 (3H, s, H-25), 1.09 (6H, s, H-26, H-30), 1.25 (3H, s, H-27), 2.05 (6H, s, 2 x OAc), 2.30 (2H, s br, H-16), 2.35 (2H, s, subm., H-22), 2.81 (1H, d, *J* = 4.7 Hz, H-12), 3.01 (1H, d, *J* = 4.7 Hz, H-11), 4.54 (2H, dd, *J* = 7.5, 8.5 Hz, H-1, H-3), 5.56 (1H, d, *J* = 5.5 Hz, H-15). ^{13}C-NMR: δ_C 16.5 (C-25), 27.5 (C-26), 29.7 (C-2), 29.9 (C-28), 31.1 (C-7), 30.3 (C-27), 32.1 (C-20), 33.5 (C-29), 35.8 (C-16), 35.9 (C-17), 36.5 (C-13), 38.1 (C-10), 39.8 (C-8), 41.1 (C-22), 41.2 (C-4), 42.5 (C-19), 48.5 (C-18), 52.6 (C-9), 53.4 (C-11), 54.6 (C-5), 58.4 (C-12), 80.6 (C-1, C-3), 118.8 (C-15), 157.2 (C-14), 170.5 ($OCOCH_3$), 170.6 ($OCOCH_3$), 215.2 (C-21).

Friedelin (**4**)

Colourless needles ($CHCl_3$), m.p. 261°C, EIMS *m/z* 426.3837 (calc. for $C_{30}H_{50}O$), 426 ([M^+], 411([M^+-CH3], 302, 273, 246, 231, 218, 205, 191, 163, 149, 137, 125, 123, 109, 95, 81. IR: ν_{max} 2930, 2870, 1716, 1460, 1388, 1188, 1110, 1074cm^{-1}. ^{1}H-NMR (500 MHz, $CDCl_3$) : δ 1.92 (1H,m, H-1_a), 1.69 (1H,m, H-1_b), 2.20 (1H,m, H-2_a), 2.21 (1H,m, H-2_b), 2.14 (1H,q, H-4), 1.50 (1H,m, H-6_a), 1.12 (1H,m, H-6_b), 1.38 (1H,m, H-7_a), 1.32 (1H,m, H-7_b), 1.35 (1H,m, H-8), 1.42 (1H,m, H-10), 1.34(1H,m, H-11_a), 1.17(1H,m, H-11_b), 1.35 (1H,m, H-12_a), 1.22(1H,m, H-12_b),1.42(1H,m, H-15_a),1.21 (1H,m, H-15_b), 1.45 (1H,m, H-16_a), 1.20 (1H,m, H-16_b), 1.49 (1H,m, H-18), 1.27 (1H,m, H-19_a), 1.11 (1H,m, H-19_b), 1.39 (1H,m, H-21_a), 1.35 (1H,m, H-21_b), 1.20 (1H,m, H-22_a), 0.96 (1H,m, (H-22_b), 0.79 (3H,d, *J* = 6.8 Hz, H-23),0.69 (3H,s, H-24), 0.85 (3H,s, H-25), 0.94 (3H,s, H-26), 0.97 (3H,s, H-27), 1.10 (3H,s, H-28), 0.96 (3H, s, H-29), 0.84 (3H,s, H-30). ^{13}C-NMR (125 MHz, $CDCl_3$): δ 41.3 (C-6), 18.6 (C-7), 52.8 (C-8), 37.1 (C-9),

60.1 (C-10), 36.0 (C-11), 29.9 (C-12), 40.1 (C-13), 39.0 (C-14), 33.1 (C-15), 35.7 (C-16), 29.4(C-17), 43.3 (C-18),34.7(C-19), 27.8 (C-20), 33.0 (C-21), 40.0 (C-22), 6.4 (C-23), 14.2 (C-24), 17.6 (C-25), 20.5(C-26), 17.9 (C-27), 31.6 (C-28), 31.9 (C-29), 33.7 (C-30).

Friedelinol (**5**)

Colourless needles, m.p. 285°C, EIMS: *m/z* 428.7337 (calculated for $C_{30}H_{52}O$, 428.7333)., 429(M+1), 414, 276, 207, 205.cm^{-1}. ^{1}H-NMR (500 MHz, $CDCl_3$) : δ 1.91 (1H, m, H-1_a), 1.67 (1H,m, H-1_b), 2.32 (1H,m, H-2_a), 2.22(1H,m, H-2_b), 3.42 (1H, dd, *J*=7.1, 3.1 Hz, H-3), 2.16 (1H,q, H-4), 1.67 (1H,m, H-6_a), 1.24 (1H,m, H-6_b), 1.48 (1H,m, H-7_a), 1.38 (1H,m, H-7_b), 1.39 (1H,m, H-8), 1.46 (1H,m, H-10), 1.30(1H,m, H-11_a), 1.16(1H,m, H-11_b), 1.28 (1H,m, H-12_a), 1.20(1H,m, H-12_b),1.41(1H,m, H-15_a),1.22 (1H,m, H-15_b), 1.47 (1H,m, H-16_a), 1.21 (1H,m, H-16_b), 1.46 (1H,m, H-18), 1.29 (1H,m, H-19_a), 1.11 (1H,m, H-19_b), 1.38 (1H,m, H-21_a), 1.31 (1H,m, H-21_b), 1.42 (1H,m, H-22_a), 0.87 (1H,m, H-22_b), 0.93(3H,d, *J* = 6.4 Hz, H-23), 0.84 (3H,s, H-24), 0.91 (3H,s, H-25), 0.95 (3H,s, H-26), 0.99 (3H,s, H-27), 1.15 (3H,s, H-28), 0.97 (3H, s, H-29), 0.84 (3H,s, H-30). ^{13}C-NMR: δ 16.02(C-1), 36.10(C-2), 72.1(C-3), 50.01(C-4), 38.67(C-5), 42.05(C-6), 18.0(C-7), 53.54(C-8), 36.90(C-9), 61.59 (C-10), 35.43(C-11),30.78(C-12), 38.0(C-13), 38.43(C-14), 32.0(C-15), 36.10(C-16), 29.90(C-17), 43.0(C-18), 35.41 (C-19), 28.43(C-20),33.02(C-21), 40.0(C-22), 12.01 (C-23), 16.67(C-24), 18.41(C-25), 18.87(C-26), 19.98(C-27), 31.92(C-28), 31.67(C-29), 35.15(C-30).
